# Minocycline Reduces Hypothalamic Microglia Activation and Improves Metabolic Dysfunction in High Fat Diet-Induced Obese Mice

**DOI:** 10.3389/fphys.2022.933706

**Published:** 2022-06-16

**Authors:** Caitlin R. Coker, Melissa White, Aneesh Singal, Sarah S. Bingaman, Anirban Paul, Amy C. Arnold, Yuval Silberman

**Affiliations:** ^1^ Department of Neural and Behavioral Sciences, College of Medicine, Pennsylvania State University, Hershey, PA, United States; ^2^ Department of Biochemistry and Molecular and Cellular Biology, Georgetown University School of Medicine, Washington, DC, United States; ^3^ Department of Comparative Medicine, College of Medicine, Pennsylvania State University, Hershey, PA, United States

**Keywords:** insulin, glucose, neuroimmune, RNAseq, paraventricular nucleus

## Abstract

Obesity is associated with insulin resistance, glucose intolerance, inflammation, and altered neuronal activity in brain regions controlling metabolic functions including food intake, energy expenditure, and glucose homeostasis, such as the hypothalamus. In this study, we tested the hypothesis that inhibiting inflammation with minocycline could reduce adverse metabolic consequences associated with high-fat diet (HFD)-induced obesity in mice and sought to determine if metabolic improvements were associated with reduced hypothalamic microglia activity. Male C57Bl/6J mice were placed on 60% HFD for 12 weeks, with minocycline (40 mg/kg, p.o.) or normal tap water given during the last 6 weeks of diet. Age-matched mice maintained on control diet were used as an additional comparator group. Metabolic function was assessed during the last week of treatment. Ramified (resting) and non-ramified (active) microglia were quantified in the hypothalamus following immunohistochemical staining of ionized calcium-binding adaptor 1 (Iba-1) and further assessed by RNAseq. In HFD fed mice, minocycline attenuated body mass and adiposity without altering food intake suggesting enhanced energy expenditure. Minocycline also attenuated hyperinsulinemia and improved insulin sensitivity in HFD mice. Increased microglial activation and autophagy gene network changes were observed in the paraventricular nucleus (PVN) of the hypothalamus of HFD mice, which was prevented by minocycline treatment. Contrary to PVN findings, there were no significant effects of either HFD or minocycline on microglia activation in the hypothalamic arcuate nucleus or central amygdala. Together, these findings suggest that minocycline improves HFD-induced weight gain and insulin resistance in part by reducing inflammatory processes in the PVN, a key hypothalamic region regulating metabolic function.

## Introduction

Obesity currently affects more than one-third of the United States population and is a leading risk factor for the development of type II diabetes and cardiovascular disease. While it is well known that obesity can lead to the development of insulin resistance and hyperglycemia, the precise link between obesity and dysregulated glucose homeostasis has yet to be fully determined. Obesity is associated with chronic low-grade inflammation within peripheral tissues, with adipose tissue being the most sensitive to obesity-induced inflammation (Lee and Lee, 2014). In particular, pro-inflammatory cytokines can reduce transcription of insulin signaling pathway components, such as insulin receptor substrate-1 (IRS-1) and glucose transporter 4 (Glut4) in adipocytes. This serves as a potential mechanism by which obesity, via pro-inflammatory cytokine upregulation, can reduce insulin sensitivity and glucose uptake in peripheral tissues such as adipose.

In addition to the periphery, obesity induces inflammation centrally in many brain regions in both humans and animal models, with recent reports indicating increased activation of hypothalamic microglia cells, the resident immune surveillance cells in the central nervous system, as being a critical target of obesity-related inflammation (Valdearcos et al., 2015). The hypothalamus contains numerous sub-regions involved in the control of energy balance, food intake, and insulin sensitivity, of which the arcuate (ARC) and paraventricular (PVN) nuclei have been most predominantly studied. Studies in the ARC have shown that microglia activation is critical for altering energy balance and inducing weight gain during long-term high-fat diet (HFD) exposure in mice, leading to enhanced susceptibility to obesity (Valdearcos et al., 2017). Recent studies have also shown that HFD exposure increases microglia activity in the PVN, and activity of inflammatory processes in this brain region is associated with alterations in adiposity, energy balance, and glucose homeostasis (de Kloet et al., 2014; Douglass et al., 2017). Together, the above-mentioned literature indicates that both peripheral and central inflammatory signaling is of critical importance in obesity-related metabolic derangements including development of insulin resistance and energy imbalance.

A previous study showed that pharmacological microglial inhibition, with the CSF1R inhibitor PLX5622, decreases HFD intake, body weight, and microgliosis (Valdearcos et al., 2017). This paper also showed that genetic modulation of microglial proinflammatory signaling altered glucose tolerance, with no assessment of insulin sensitivity, in lean mice. To our knowledge, no studies have examined if pharmacologic inhibition of microglial activation with minocycline can reduce neuroinflammation as well as improve insulin and glucose homeostasis in mouse models of diet-induced obesity. Since microglia are thought to be the primary regulators of innate immune capacity, especially centrally (DiSabato et al., 2016), we sought to test the hypothesis that microglia inhibition could improve glucose homeostasis and energy balance in diet-induced obesity. To test this, we utilized minocycline, a tetracycline antibiotic that is able to cross the blood brain barrier and demonstrates anti-inflammatory capabilities via microglial inhibition, as a tool to dissect the contribution of inflammation to disturbances in glucose homeostasis and energy balance in HFD-induced obese mice.

## Materials and Methods

### Approvals

The Institutional Animal Care and Use Committee at the Pennsylvania State University College of Medicine approved all procedures. Macroenvironmental conditions and procedures were in compliance with the NIH Guide for the Care and Use of Laboratory Animals.

### General Protocol

Male 5-week-old C57BL/6J mice (Jackson Laboratory; Bar Harbor, ME) were group housed on a 12:12 h light cycle with controlled humidity and temperature maintained at approximately 23°C. Mice were weight-matched and placed on 60% HFD (60% calories from fat, 26% calories from carbohydrates, 14% calories from protein; Bioserv F3282; Flemington, NJ) ad libitum. After 6 weeks on control or HFD, mice were acclimated to individual cages, maintained on their assigned *ad libitum* diet, and given tap water or water treated with minocycline (40 mg/kg/day) for 6 weeks. Water bottles were changed twice per week. While minocycline does effectively cross the blood-brain barrier, most animal and human studies employ a systemic route of administration. We chose to give minocycline in the drinking water as it more closely mimics oral ingestion in humans, which is the most common route of administration clinically. Body mass was measured weekly. During the last week of treatment, body composition was measured, and insulin and glucose tolerance tests were performed while continuing respective diet and drug treatments. Water and food intake were also measured during this period and reported as a 24-h average intake at the end of treatment. An additional cohort of mice were age and weight matched at the start of study and maintained on a control diet (16% calories from fat, 63% calories from carbohydrates, 21% calories from protein; Bioserv F4031; Flemington, NJ) to serve as a comparator group to assess the impact of HFD on metabolic and inflammatory outcomes.

### Body Composition

Nuclear magnetic resonance imaging was used to measure fat, lean, and fluid masses in conscious mice (Bruker LF110 Minispec; Billerica, MA), with values reported as percentages of total body mass.

### Insulin and Glucose Tolerance Tests (ITT and GTT)

As recently published by our group (White et al., 2019; Coker et al., 2020), for ITT, mice were fasted 4 h and then injected intraperitoneally with insulin (0.75 units/kg of regular U-100 insulin in 1x PBS; Novolin; Plainsboro, NJ). A tail vein blood sample was taken at baseline (immediately before injection) and at 15, 30, 60, 90, and 120 min post-insulin injection to measure blood glucose with a glucometer (Prodigy AutoCode; Charlotte, NC). An additional blood sample was taken at baseline with a micro-hematocrit capillary tube (FisherBrand; Waltham, MA, United States) for measurement of plasma insulin concentration. For GTT, mice were fasted overnight and then injected intraperitoneally with dextrose (2 g/kg of 50% dextrose; Hospira, Inc.; Lake Forest, IL, United States). Blood glucose was measured at baseline (immediately before injection) and at 15, 30, 60, 90, and 120 min post-dextrose injection. Plasma insulin concentration was determined at baseline and at 15 and 120 min post-injection. At least 2 days were allowed for recovery between ITT and GTT procedures. Plasma insulin concentration was measured using a mouse ultrasensitive insulin ELISA (Alpco; Salem, NH).

### Euthanasia and Tissue Collection

At the end of studies, a subset of mice was fasted for 4 h and then euthanized under isoflurane anesthesia (*n* = 5 Control, *n* = 5 HFD and *n* = 8 HFD+Mino). Epididymal visceral white adipose (EPF), inguinal subcutaneous white adipose (SCF), and interscapular brown adipose (BAT) tissues were collected, weighed, flash frozen, and stored at -80°C for gene expression analysis. The remaining mice (*n* = 7 Control, *n* = 4 HFD and *n* = 9 HFD+Mino) were anesthetized with isoflurane and transcardially perfused with 0.01 M phosphate-buffered saline (PBS; 15 ml) followed by 4% paraformaldehyde in PBS (20 ml). Brains were removed, post-fixed for 24 h in 4% paraformaldehyde/PBS fixative solution at 4°C, and then placed in 30% sucrose/PBS solution for an additional 48 h at 4°C. Coronal sections (40 μm) were cut with a cryostat (Microm HM550, Thermo Scientific; Waltham, MA, United States) and stored at 4°C in cryoprotectant prior to immunofluorescence staining.

### Immunofluorescence Staining

Free-floating brain slices containing ARC and PVN were washed in 0.01 M PBS (4 × 10 min), permeabilized in 0.5% Triton X-100/PBS solution (30 min), and blocked with 10% Normal Donkey Serum in a 0.1% Triton X100/PBS solution (1 h). Goat anti-Iba1 antibody (1:1000, ab5076, Lot GR254159-5, Abcam; Cambridge, MA) was added directly to the blocking solution and incubated for 72 h at 4°C. After this incubation period, sections were washed in PBS (4 × 15 min) and subsequently transferred to a 0.1% Triton X-100/PBS solution containing 10% Normal Donkey Serum and donkey anti-goat Alexa Fluor 568 fluorescent conjugated secondary antibody (1:500, Lot 1711491, Invitrogen; Eugene, OR) for 24 h at 4°C. Sections were washed in PBS (4 × 10 min), mounted onto slides with ProLong Gold Mounting media (Lot 1887458, Invitrogen; Eugene, OR, United States), and allowed to air-dry overnight. All steps were performed on a nutating mixer at room temperature unless otherwise noted.

### Imaging and Image Analysis

An Olympus IX81 scanning confocal microscope was used to capture z-stack images of stained ARC and PVN sections. ImageJ software was used to analyze stacked images. Microglia were classified into ramified (resting microglia) and non-ramified (active microglia) using previously established methods (Cerbai et al., 2012). Resting microglia were characterized by small, round cell bodies that displayed many long, thin processes. Active microglia were identified by large, darkly stained cell bodies that lacked ramification or displayed shorter, twisted processes. All Iba1-positive cells within the image frame were quantified and “percent active” indicates number of cells identified as active divided by total number of Iba1 positive cells. Images were de-identified and analyzed by two independent researchers blinded to treatment condition, with their results within 1% error, and final cell counts for each brain region averaged per mouse (at least two images per brain region per mouse).

### Gene Expression

PVN, ARC, and central amygdala were isolated via microdissection utilizing the brain punch technique and immediately frozen. Frozen tissue was homogenized in QIAzol Lysis Reagent using a TissueLyser II, with total RNA extracted using RNAeasy Lipid Tissue Mini kits and QIACube automated processing (Qiagen; Germantown, MD, United States). RNA concentration was measured with a NanoDrop spectrophotometer (ND-1000, Thermo Fisher Scientific; Waltham, MA, United States). cDNA was synthesized from total RNA using a high-capacity cDNA reverse transcription kit (ThermoFisher Scientific; Waltham, MA, United States). Quantitative real-time polymerase chain reaction (qPCR) was performed on a QuantStudio 12K Flex system (Applied Biosystems; Foster City, CA, United States) using mouse specific Taqman gene primers (ThermoFisher Scientific; Waltham, MA, United States). The primers used were: interleukin 6 (IL6; mm00446190_m1), tumor necrosis factor α (TNFα; mm00443258_m1), interleukin 1β (IL1β; mm00434228_m1), and interleukin 10 (IL10mm01288386_m1). Each sample was measured in triplicate with cycle threshold (CT) values normalized to 18S ribosomal RNA (Rn18s, mm03928990_g1). Relative gene expression was determined using the 2^–∆∆CT^ method.

### RNA Extraction for RNAseq

A small cohort of control, HFD, and HFD+Mino mice (*n* = 3 independent mice per group) underwent the same diet exposure as the mice in the above groups but did not undergo metabolic testing. At the end of study, mean body mass was not different between these mice and their respective groups in the other cohorts described above. PVN tissue punches were then utilized for RNAseq. Approximately 30–60 mg of tissue sample was transferred to a safe-lock microcentrifuge tube (Eppendorf). A mass of stainless-steel beads (Next Advance, cat# SSB14B) equal to the mass of the tissue was added to the tube. Two volumes of TRI Reagent^®^ (Zymo Research) were added to the tube and samples were immediately mixed in a bead mill homogenizer (Bullet Blender, Next Advance) for 1 min at a speed of ten. Samples were visually inspected to confirm desired homogenization and then incubated at 37°C for 5 min. The TRI Reagent^®^ was added up to 0.6 ml, and samples were mixed in the Bullet Blender for 1 min. Total RNA was extracted using Direct-zol™ RNA Miniprep Kit (Zymo Research). Optical density values of extracted RNA were measured using NanoDrop (Thermo Scientific) to confirm an A260:A280 ratio above 1.9. RNA integration number (RIN) was measured using BioAnalyzer (Agilent Technologies) RNA 6000 Nano Kit.

### Library Preparation and Sequencing for Bulk mRNA

The cDNA libraries were prepared using the SMARTer Ultra Low Input RNA Kit for Sequencing—v4 (TAKARA Bio) and Nextera XT DNA Library Prep Kit (Illumina) as per manufacturer’s instructions. The unique barcode sequences were incorporated in the adaptors for multiplexed high-throughput sequencing. The final product was assessed for its size distribution and concentration using BioAnalyzer High Sensitivity DNA Kit (Agilent Technologies). The libraries were pooled and diluted to 3 nM using 10 mM Tris-HCl, pH 8.5 and then denatured using the Illumina protocol. The denatured libraries were loaded onto an S1 flow cell on an Illumina NovaSeq 6000 (Illumina) and run for 2 × 50 cycles according to the manufacturer’s instructions. De-multiplexed sequencing reads were generated using Illumina bcl2fastq (released version 2.18.0.12) allowing no mismatches in the index read.

### RNA Sequencing (RNASeq) Data Analysis

The samples from Control, HFD, and HFD+Mino groups (*n* = 3 independent mice per group) were assessed in triplicate. RNA sequence reads were aligned to the mouse genome, mm 10, using HISAT2 (Kim et al., 2019); mapped reads were assigned to genomic features using featureCounts (Liao et al., 2014), and differential expression analysis of RNA-Seq expression profiles were conducted using edgeR (Robinson et al., 2009; McCarthy et al., 2012). Boxplot and heatmaps were plotted using R-packages easyGgplot2 and gplots. Pathway analysis was conducted using WebGestalt tool (Liao et al., 2019) and KEGG pathway database. Figures were made using Adobe Illustrator.

### Statistical Analysis

Data were analyzed and graphed using GraphPad Prism (GraphPad Software, versions 8 and 9; San Diego, CA, United States) and Microsoft Excel 365 (Microsoft Corporation; Redmond, WA, United States). Metabolic data were analyzed using one-way ANOVA with Tukey’s post-hoc analysis. Analysis of covariance (ANCOVA) was used to determine the influence of body mass on food and water intake among groups. Data are represented as mean ± standard error of the mean (SEM). Significance was determined at the *p* < 0.05 level for all analyses.

## Results

### Minocycline Attenuates Weight Gain and Body Composition Changes Under High Fat Diet Conditions

**FIGURE 1:**
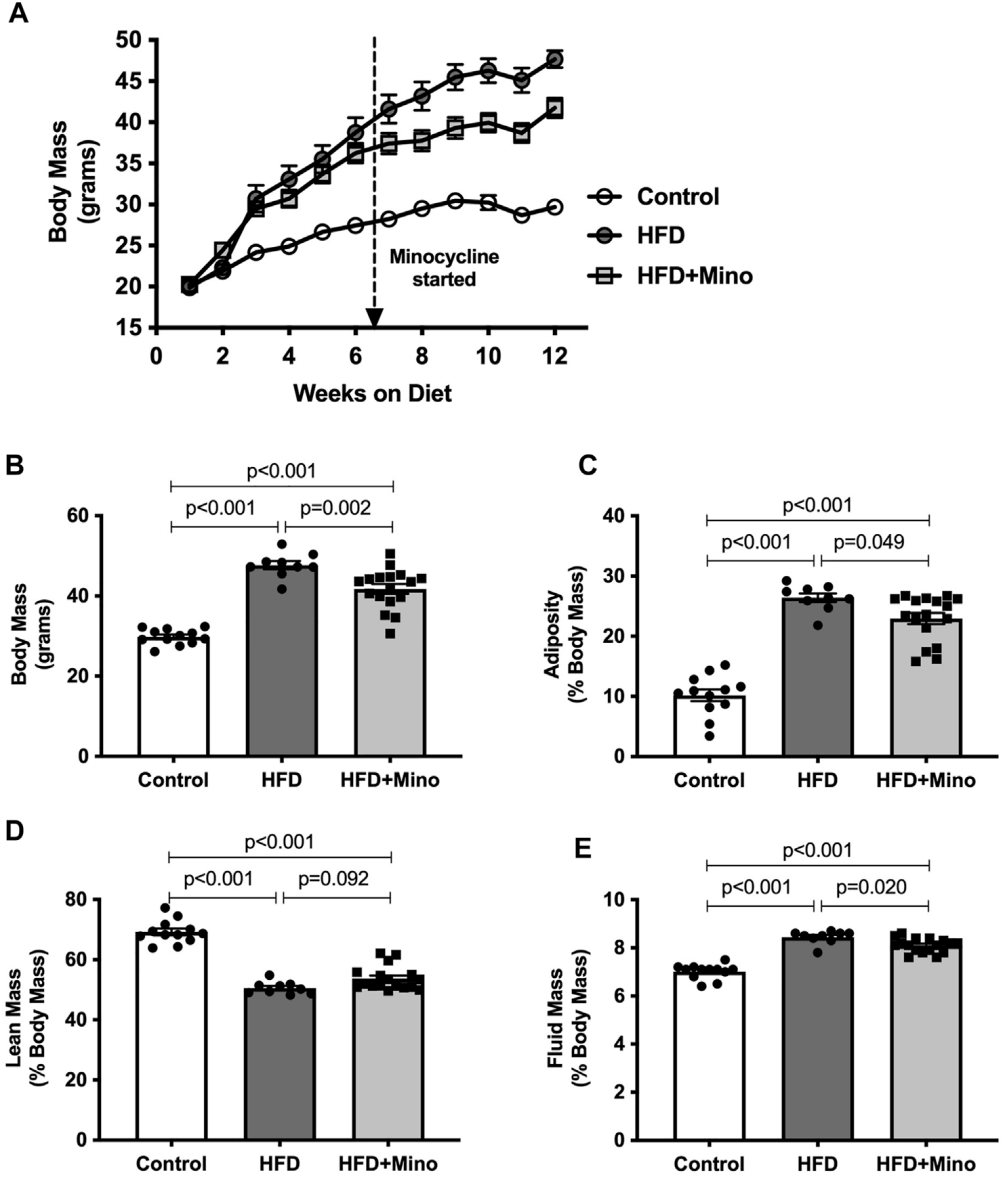
Chronic minocycline treatment significantly attenuates HFD-induced weight gain. Body mass was measured weekly and body composition at end of treatment in control diet (Control; *n* = 12), high fat diet (HFD; *n* = 9), and HFD mice treated with minocycline (HFD+Mino; *n* = 17). **(A,B)** Minocycline attenuated the HFD-induced increase in body mass, which was associated with a small reduction in adiposity **(C)** and fluid mass **(E)** and a trend toward an increase in lean mass **(D)**. Data were analyzed by one-way ANOVA with Tukey’s post-hoc analysis; *p* < 0.05.

As expected, HFD mice exhibited increased body mass throughout the study compared to control diet mice. Although body mass was not different between HFD and HFD+Mino mice at 6 weeks of study prior to minocycline treatment, weight gain was attenuated in HFD+Mino mice following initiation of minocycline treatment (Figure 1A). At the end of treatment, body mass was significantly higher in HFD (47.7 ± 1.0 g) compared to Control (29.8 ± 0.6 g) and HFD+Mino (41.8 ± 1.2 g) groups (F_(2,35)_ = 61.69, *p* < 0.001 one-way ANOVA; Figure 1B). The attenuated weight gain in HFD+Mino mice was associated with reduced adiposity (F_(2,35)_ = 72.72, *p* < 0.001 one-way ANOVA; Figure 1C) and fluid mass (F_(2,35)_ = 75.53, *p* < 0.001 one-way ANOVA; Figure 1E) with increased lean mass (F_(2,35)_ = 90.40, *p* < 0.001 one-way ANOVA; Figure 1D).

### Minocycline Does Not Alter Food and Water Intake in High Fat Diet Mice

**FIGURE 2:**
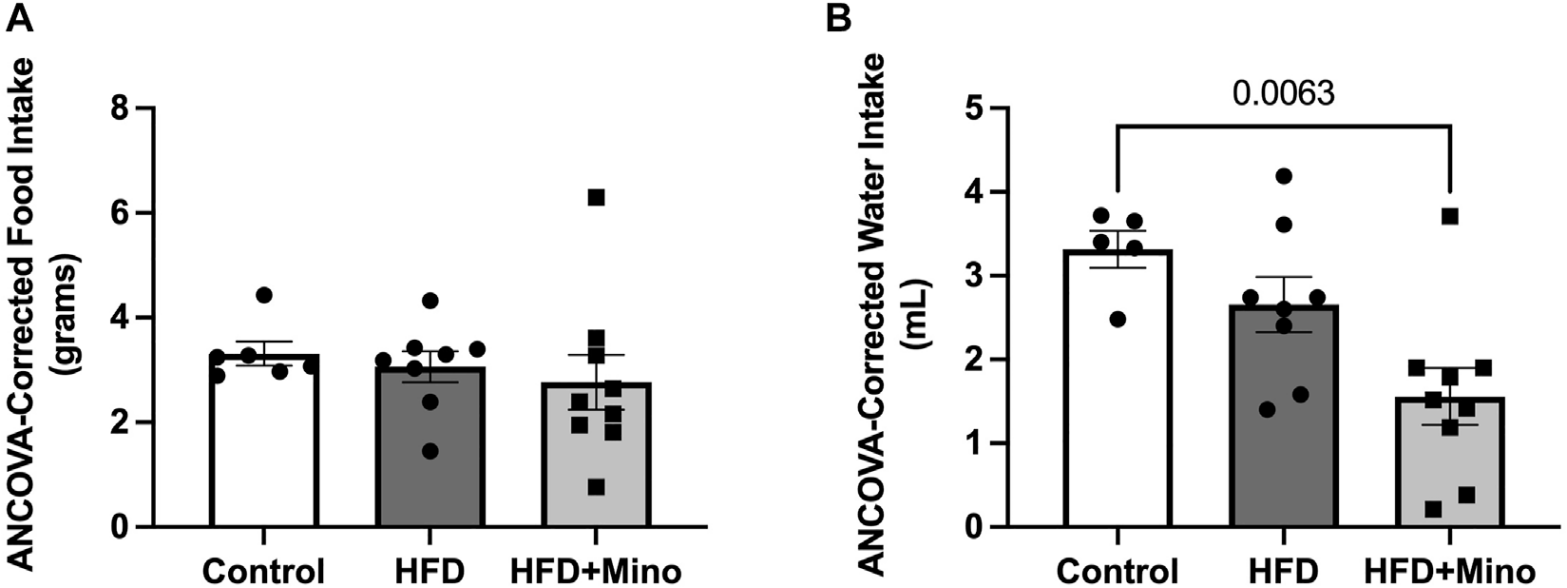
Minocycline does not alter HFD-induced changes in food and water intake. Food and water consumption were assessed over a 24-h period in a subset of control diet (Control; *n* = 6), high fat diet (HFD; *n* = 7), and HFD mice treated with minocycline (HFD+Mino; *n* = 15) during the last week of study. These measurements were corrected for body mass via analysis of covariance (ANCOVA). **(A)** Grams of food consumed did not differ among groups. **(B)** HFD and HFD+Mino groups consumed less milliliters of water compared to Control.

Minocycline is an antibiotic that has previously been shown to reduce food and water intake in animals due to potential effects on gastrointestinal function. To test for any possible confounding effects of minocycline on food or water intake in our study, ANCOVA was used to compare food and water intake during the last week of study, corrected for body mass. There was no significant difference in grams of food intake per day among groups (Figure 2A, *p* = 0.116). HFD mice consumed less milliliters of water per day compared to Control (Figure 2B, F_(2,35)_ = 7.89, *p* = 0.001), but water consumption was not significantly different between HFD and HFD+Mino groups.

### Minocycline Restores Insulin Sensitivity in High Fat Diet Mice

**FIGURE 3:**
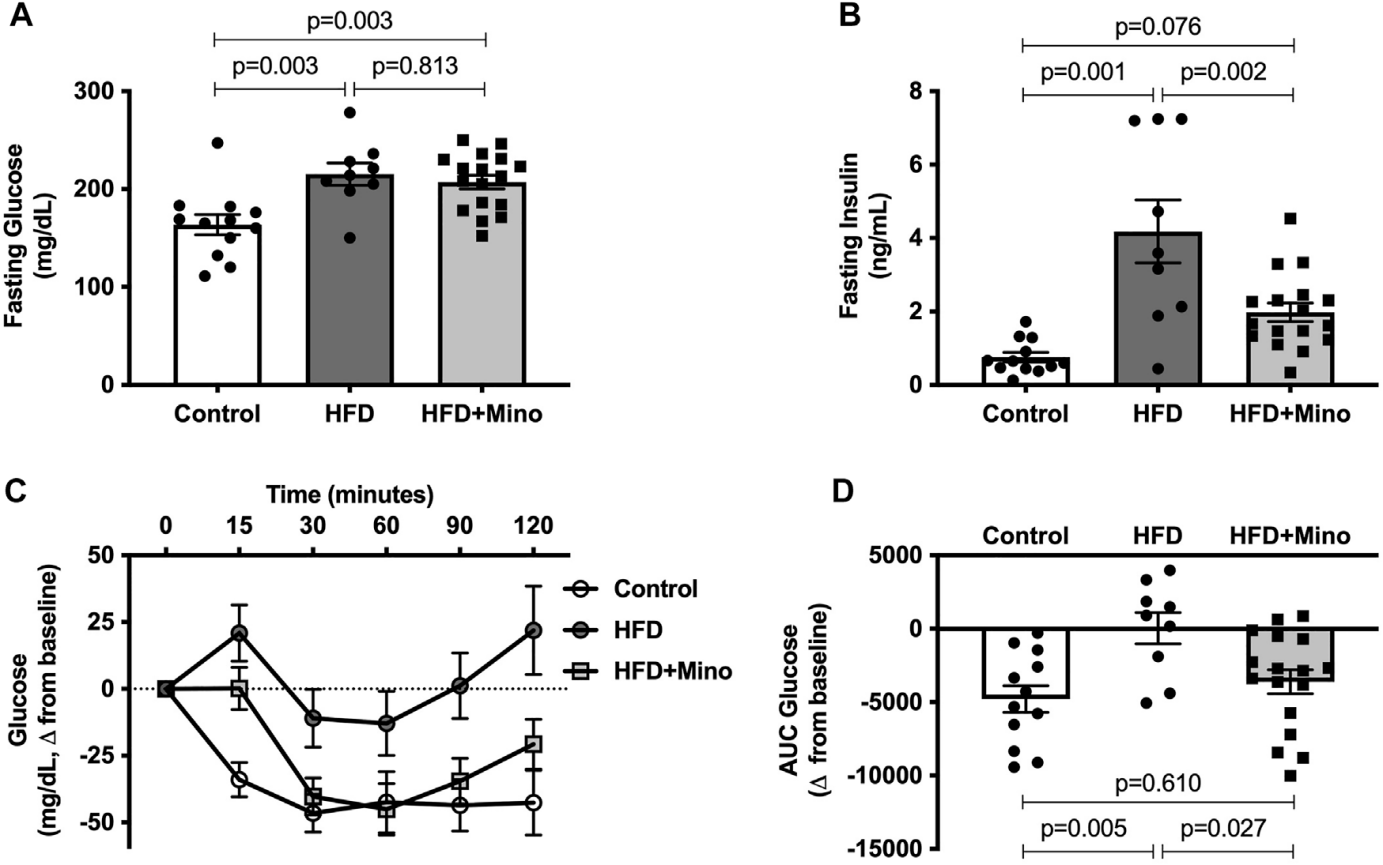
Minocycline attenuates hyperinsulinemia and improves insulin sensitivity in HFD mice. Following a 4-h fast, insulin tolerance tests were performed at end of treatment in control diet (Control; *n* = 12), high fat diet (HFD; n = 9), and HFD mice treated with minocycline (HFD+Mino; *n* = 17). **(A,B)** Minocycline reduced elevated fasting insulin levels in HFD mice, without effects on fasting glucose. **(C,D)** HFD reduced insulin sensitivity compared to Control, which was restored with minocycline treatment. Data were analyzed by one-way ANOVA with Tukey’s post-hoc analysis; *p* < 0.05.

Prior to ITT, blood samples were taken for measurement of fasting glucose and insulin levels. One-way ANOVA (F_(2,35)_ = 8.60, *p* < 0.001; Figure 3A) followed by Tukey’s post-hoc analysis indicated that 4-h fasting glucose was elevated in HFD (215 ± 11 mg/dl) and HFD+Mino (207 ± 7 mg/dl) groups compared to Control (164 ± 10 mg/dl), with no difference between HFD and HFD+Mino groups (*p* = 0.813). The hyperinsulinemia observed in HFD mice (4.2 ± 0.9 ng/ml) was attenuated with minocycline treatment (2.0 ± 0.2 ng/ml), towards levels seen in Control mice (0.8 ± 0.1 ng/ml) (one-way ANOVA, F_(2,35)_ = 14.72, *p* < 0.001; Figure 3B). Given that maintenance of glycemia with reduced insulin levels in HFD+Mino mice is suggestive of improved insulin sensitivity, we performed an ITT. The change in blood glucose levels from baseline in response to exogenous insulin administration is shown in Figure 3C. The area under the curve (AUC) for change in blood glucose levels is shown in Figure 3D, with a more negative value indicating greater insulin sensitivity. One-way ANOVA (F_(2,35)_ = 5.97, *p* = 0.006; Figure 3D) followed by Tukey’s post-hoc analysis indicated HFD mice (44 ± 1066 glucose mg/dL*min) had significantly reduced insulin sensitivity compared to Control mice (-4781 ± 914 glucose mg/dL*min), which was restored with minocycline treatment (-3,606 ± 816 glucose mg/dL*min).

### Minocycline did Not Alter Glucose Tolerance

**FIGURE 4:**
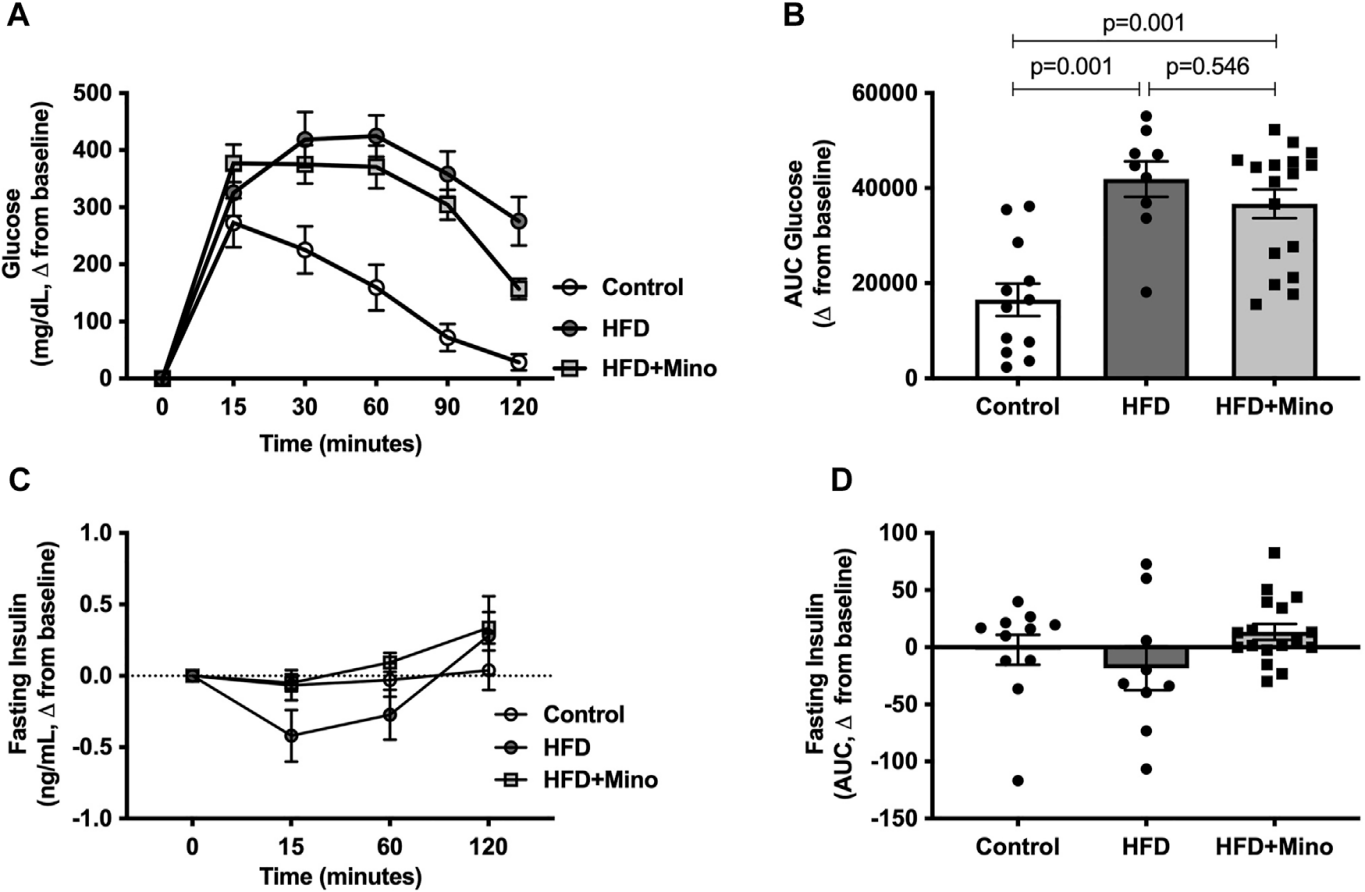
Minocycline does not improve HFD-induced glucose intolerance. Following an overnight fast, glucose tolerance tests were performed at the end of treatment in control diet (Control; *n* = 12), high fat diet (HFD; *n* = 9), and HFD mice treated with minocycline (HFD+Mino; *n* = 17). **(A,B)** HFD fed mice developed glucose intolerance, which was not corrected by minocycline treatment. **(C,D)** There was no difference in plasma insulin levels over the 120-min study period suggesting lack of effect of minocycline on glucose-stimulated endogenous insulin secretion. Data were analyzed by one-way ANOVA with Tukey’s post-hoc analysis; *p* < 0.05.

The change in blood glucose levels from baseline in response to exogenous dextrose administration is shown in Figure 4A. The AUC for change in blood glucose levels is shown in Figure 4B, with a more positive value indicating glucose intolerance. One-way ANOVA (F_(2,35)_ = 14.40, *p* < 0.001; Figure 4B) followed by Tukey’s post-hoc analysis indicated glucose intolerance in HFD (41,918 ± 3,731 glucose mg/dL*min) and HFD+Mino (36,696 ± 3,012 glucose mg/dL*min) groups compared to Control (16,522 ± 3,420 glucose mg/dL*min), with no difference between HFD and HFD+Mino. Similarly, there were no differences in glucose-stimulated endogenous insulin secretion (as indicated by the AUC for fasting insulin levels over the 120-min study period) among groups (Figures 4C,D).

### Minocycline did Not Alter Obesity-Related Peripheral Adipose Inflammation

**FIGURE 5:**
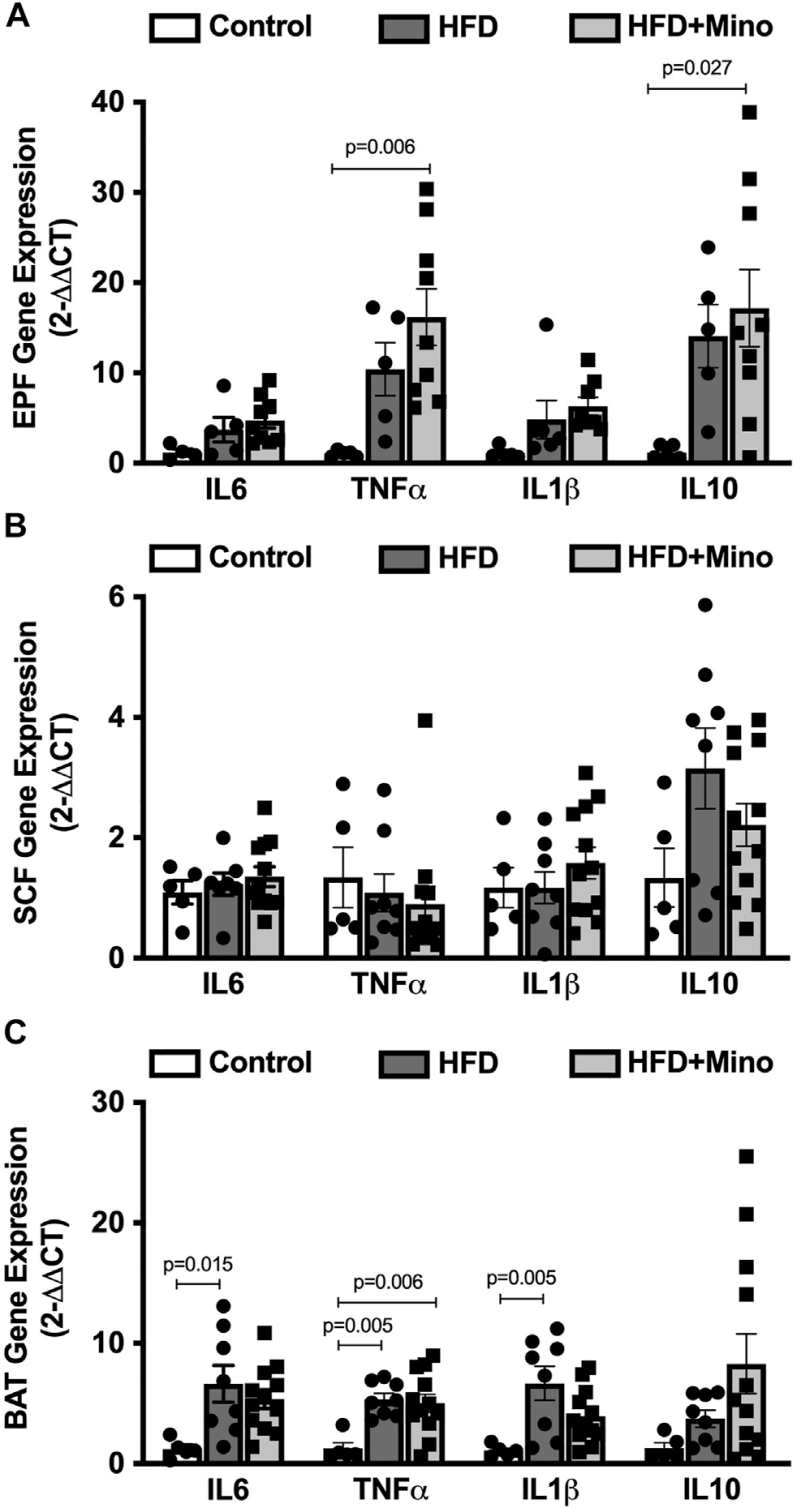
Minocycline does not alter HFD-induced adipose inflammation. Gene expression of inflammatory cytokines was determined in epididymal visceral white adipose (EPF), inguinal subcutaneous white adipose (SCF), and brown adipose (BAT) tissues at the end of treatment in control diet (Control; *n* = 5), high fat diet (HFD; *n* = 5), and HFD mice treated with minocycline (HFD+Mino; *n* = 8). **(A)** HFD increased gene expression of tumor necrosis factor α (TNFα) and interleukin 10 (IL10), and minocycline further increased mRNA of these cytokine markers in EPF. **(B)** There were no differences in gene expression of inflammatory markers in SCF among groups. **(C)** Gene expression of the pro-inflammatory markers interleukin 6 (IL6), TNFα, and interleukin 1β (IL1β) was upregulated in BAT in HFD mice, with no effect of minocycline on these markers. Data were analyzed by one-way ANOVA with Tukey’s post-hoc analysis; *p* < 0.05.

We next determined the effects of minocycline treatment on markers of inflammation in adipose tissue. Gene expression of pro- and anti-inflammatory cytokines was determined in visceral (EPF), subcutaneous (SCF), and brown (BAT) adipose tissues. In EPF, HFD increased mRNA of the pro-inflammatory cytokine TNFα and anti-inflammatory cytokine IL10, with an even greater upregulation observed following minocycline treatment (Figure 5A). In SCF, there were no differences in mRNA for inflammatory genes among groups (Figure 5B). In BAT, HFD increased mRNA of the pro-inflammatory cytokines IL6, TNFα, and IL1β, with no effect of minocycline on these markers (Figure 5C).

### Minocycline Reduces Obesity-Related Hypothalamic Neuroinflammation

**FIGURE 6:**
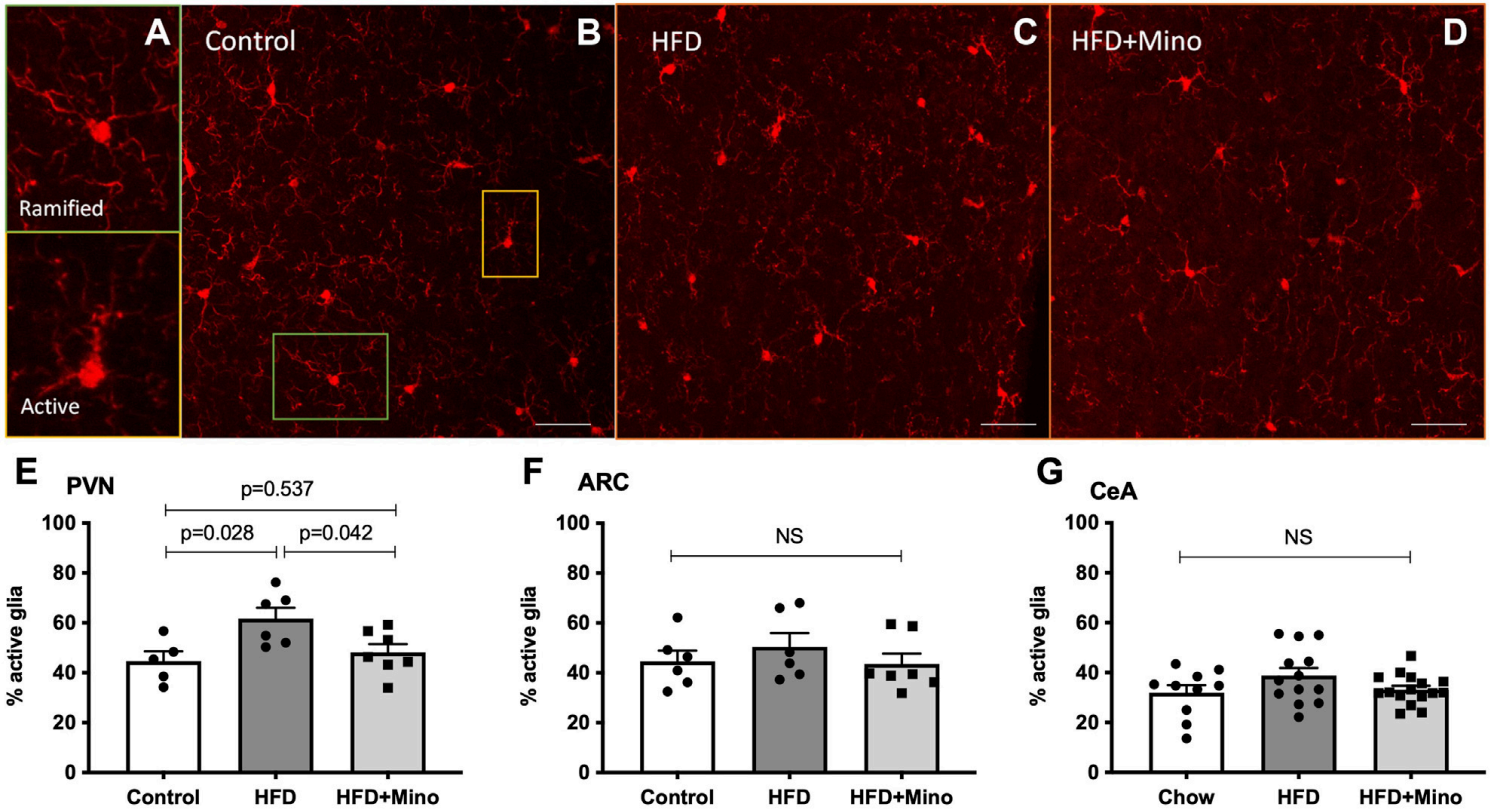
Minocycline mitigates HFD-induced increases in active microglia in the PVN. Ramified (resting) and non-ramified (active) microglia were quantified following immunohistochemical staining of Iba-1 in control diet (Control; *n* = 7), high fat diet (HFD; *n* = 4), and HFD mice treated with minocycline (HFD+Mino; n = 9). **(A)** Exemplar images of resting microglia with small, round cell body and many long, thin process (ramified) and active microglia with large, elongated cell body and few processes (non-ramified). **(B–D)** Exemplar 40x Iba-1 images from Control, HFD, and HFD+Mino groups. Scale bar = 20 μm **(E)** An increased percentage of active microglia was observed in the paraventricular nucleus (PVN) of HFD mice, which was prevented with minocycline **(F–G)** There was no significant effect of HFD or minocycline on microglia activation in the arcuate nucleus (ARC) or the central nucleus of the amygdala (CeA). Data were analyzed by one-way ANOVA with Tukey’s post-hoc analysis; *p* < 0.05.

We next determined if the metabolic improvements with minocycline were associated with reduced microglia activity in the PVN or ARC. Following the last week of treatment, mice were euthanized and examined for changes in microglia morphology via immunohistochemistry using ionized calcium binding adaptor 1 (Iba-1) antibody staining as described by (Cerbai et al., 2012) (Figures 6A–D). Ramified (resting) microglia are characterized by their small, round cell bodies and many long, thin processes while non-ramified (active) microglia are characterized by their large, elongated cell bodies and few processes (Figure 6A). One-way ANOVA (F_(2,15)_ = 5.247, *p* = 0.019; Figure 6E) indicated a significant increase in the percentage of active microglia in the PVN of HFD mice (62 ± 4%), which was decreased by minocycline treatment (48 ± 3%) to levels seen in control diet mice (45 ± 4%). There was no significant effect of either HFD or minocycline on microglia activation in the ARC (Figure 6F, One-way ANOVA F_(2,16)_ = 0.645, *p* = 0.54) or in the central nucleus of the amygdala (Figure 6G, One-way ANOVA F_(2, 7)_ = 1.039, *p* = 0.40).

### High Fat Diet and Minocycline Modulation of PVN Gene Networks

**FIGURE 7:**
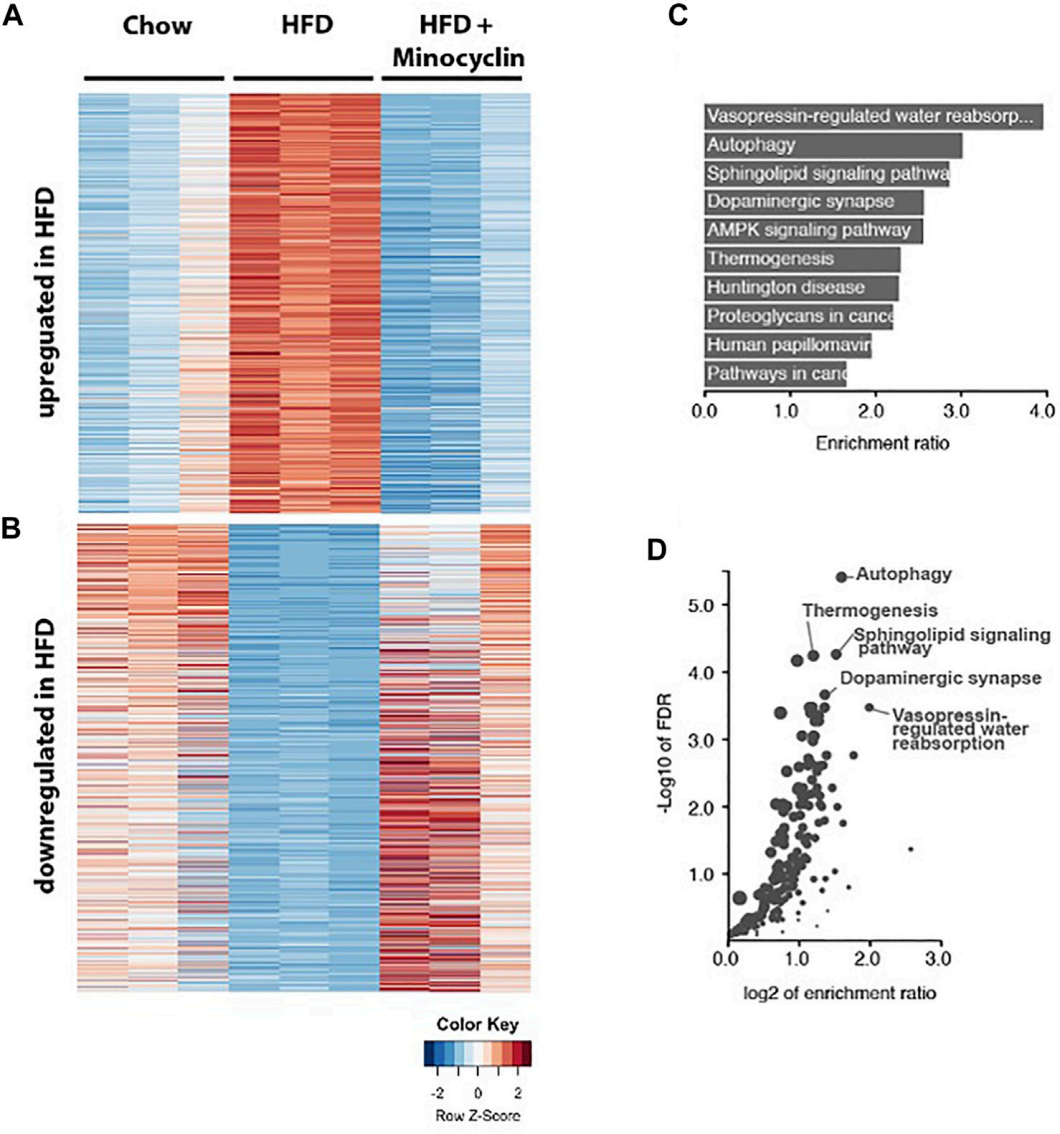
RNA sequencing of PVN identifies genes associated with HFD that are rescued by minocycline treatment. **(A,B)** Heat map showing the HFD upregulated and downregulated genes compared to control chow diet and HFD+minocycline treatment. Columns are biological replicates, rows are genes with colored scaled row z-score of log2 (cpm+1) gene expression values for each sample. **(C–D)** Pathway analysis of the HFD altered gene set in A with enrichment ratio **(C)** and FDR values **(D)** identifies autophagy as one of the highest ranked and significant pathways in the PVN.

Given that minocycline decreased morphologic measures of HFD-induce microglia reactivity in the PVN, we next sought to determine the molecular correlates of HFD-induced gene expression changes in this brain region. A 3-way differential gene expression analysis was conducted to identify significantly upregulated and downregulated genes upon HFD intake that were restored upon HFD+Mino treatment. We identified 839 upregulated and 797 downregulated transcripts that were expressed ±1.5 folds at false discovery rate (FDR) < 0.01 upon HFD diet with respect to their mean expression level across conditions (Figure 7A, Supplementary Table S1). Pathway enrichment analysis of these total 1636 up and downregulated transcripts showed the cellular autophagy pathway, critical in inflammation and microglial polarization (Levine et al., 2011; Jin et al., 2018), had the second highest enrichment ratio of 3.0 (*p* = 1.3e-8) with 31 overlapping genes (Figure 7B) and highest -Log10 FDR (Figure 7C). Within the autophagy pathway (Supplementary Figure S1), we noted the expression of a number of genes related to insulin signaling were shown to be disrupted by HFD including PI3K, PTEN, PDK1, AKT, and RAS (Supplementary Figure S2) were normalized by minocycline treatment.

## Discussion

The current study aimed to examine if inhibiting inflammation with minocycline reduces the negative metabolic consequences associated with HFD consumption in mice. We further determined if these metabolic improvements were associated with altered microglia activity and gene expression in brain regions associated with regulating metabolic function. Minocycline treatment was associated with a decrease in HFD-induced weight gain, improvement in insulin sensitivity, decrease in active microglia, and restoration of alterations in autophagy-related gene networks in the PVN of the hypothalamus. These positive metabolic effects occurred independent of changes in food or water intake and peripheral adipose inflammation. Together, these findings suggest that minocycline improves HFD-induced weight gain and insulin resistance, and this may be due, at least in part, to reducing central inflammatory processes in the PVN.

Previous studies have shown that reductions in inflammation can improve HFD-induced metabolic dysfunction but few, if any, studies have utilized a widely available and FDA-approved medication in such studies, limiting clinical applications of prior research. In this study, we utilized minocycline as a tool to determine if reducing inflammation may improve metabolic disruptions following HFD exposure. Minocycline is an FDA-approved tetracyclic antibiotic with anti-inflammatory properties at the doses utilized in the current study (Sun et al., 2015). Our findings indicate that minocycline can reduce hyperinsulinemia, improve insulin sensitivity, and attenuate HFD-induced increases in body mass and adiposity without altering food or water intake. This is similar to a previous study in which rats given minocycline following HFD exposure had improvements in body fat accumulation and body weight gain, but this was due to a decrease in food intake (Vaughn et al., 2017). Despite weight loss and restoration of insulin sensitivity, mice treated with minocycline remained glucose intolerant, which can contribute to elevated blood glucose and predispose to type II diabetes. This may, in part, explain our finding that minocycline did not improve fasting hyperglycemia. While we do not know the reason that minocycline failed to improve glucose tolerance in this study, it is important to note that glucose tolerance tests encompass multiple physiological responses in addition to insulin sensitivity such as insulin secretion, glucose uptake and effectiveness, glucose absorption and counter-regulatory hormone responses. It is possible that despite correcting insulin sensitivity, minocycline did not improve these other physiological mechanisms. In addition to metabolic dysfunction, obesity is also associated with cardiovascular complications. While we did not assess cardiovascular parameters in this study, previous work has shown minocycline lowers blood pressure in rodent models of essential hypertension (Galla et al., 2018) and patients with resistant hypertension (Pepine et al., 2021). To our knowledge, the ability of minocycline to alter blood pressure in obesity-related hypertension has not been examined and will be of interest in future studies.

Our findings suggest that minocycline also reduced HFD-induced microglia activation in the PVN, without altering peripheral inflammatory markers or microglia activation in the ARC. The reduction in microglia activation also appeared to be associated with restoration of HFD-induced alteration in autophagy pathways in the PVN. We speculate these findings suggest that beneficial effects of minocycline on HFD-induced metabolic dysfunction may be regulated, at least in part, via selective reductions in PVN neuroinflammatory processes. Although not tested here, minocycline has been suggested to modulate high fat diet outcomes via alteration of the gut microbiome (Hasebe et al., 2019; Leigh et al., 2020; Vaughn et al., 2017), which has also been shown to alter neuroinflammatory signaling (Haase et al., 2020; Rea et al., 2016; Rutsch et al., 2020).

Accumulating data support that inflammatory responses, including microglial activation, impact hypothalamic circuits controlling energy homeostasis and that the inflammatory activation state of microglia controls hypothalamic immune responses to HFD and regulates the susceptibility to obesity (Thaler et al., 2010). The precise mechanisms by which microglial activation influence energy balance are still under investigation but may include reduced leptin sensitivity as well as interactions with the sympathetic nervous system to impair adipose thermogenesis. Consistent with this concept, previous studies have also shown that microglial inhibition with minocycline reduces food consumption in HFD-fed rats (Vaughn et al., 2017). Genetic or pharmacologic depletion of microglia also resulted in a reduction in body mass and food intake in HFD-fed mice (Valdearcos et al., 2017). These previous studies contrast our finding of no differences in food intake with minocycline in HFD mice. These disparate findings could reflect differences in species (mice versus rats), length of HFD exposures (12 weeks versus 1–3 weeks), and methods to deplete microglia (oral versus intraperitoneal minocycline, transgenic deletion of microglial IKKB versus colony stimulating factor 1 receptor inhibition). Of note, these prior studies did not assess the impact of minocycline on measures of glucose homeostasis.

Together, it is possible that minocycline treatment may alter HFD consumption due to altered taste properties or alterations in brain pathways regulating food intake in these previous studies. Such reductions in food intake in previous studies would result in body mass reductions, which in itself may improve metabolic dysfunction. Our finding for reduced body weight in the absence of changes in food intake is suggestive of increases in energy expenditure, which could encompass changes in resting metabolic rate, thermic effects of food, or locomotor activity; however, energy expenditure was not measured in the current study and will need to be assessed in future studies. Minocycline given orally in rodents could also potentially alter water intake, however, we found no difference in grams of water consumed between HFD and HFD+Mino groups in the current study. Together, our findings suggest minocycline treatment did not produce taste aversions or alter food or water intake regulation and that the ability of minocycline to improve insulin sensitivity and weight gain is associated with reductions in PVN neuroinflammation. This is possible as the PVN controls autonomic projections regulating pancreatic insulin secretion, peripheral glucose uptake, and hepatic glucose flux (Hill, 2012).

HFD is known to promote inflammation in both peripheral and central tissues. In particular, HFD-induced inflammation in the hypothalamus has been extensively studied in the context of food consumption, energy expenditure, insulin and glucose homeostasis and regulates susceptibility to obesity (Thaler et al., 2012, 2010; Valdearcos et al., 2017, 2015). However, to our knowledge, this work is the first to examine how pharmacologic manipulation with an FDA-approved drug with anti-inflammation properties may impact both peripheral and central inflammation and modulate glucose homeostasis. As mentioned above, in this study, we found selective effects on inflammation in the PVN, but we did not observe a change in the number of reactive microglia in the ARC or central amygdala. As this is a targeted study, we did not assess additional brain regions or other hypothalamic subregions that are involved in insulin and glucose homeostasis and cannot rule out the possibility that microglial activation may be altered in brain regions not tested. While HFD resulted in increased proinflammatory makers in white visceral and brown adipose tissue in this study, minocycline did not impact these markers of peripheral inflammation. This is consistent with a previous study showing that minocycline can reduce inflammatory gene expression in the hippocampus without impacting white adipose inflammatory signaling in a high-fat, high-sugar cafeteria diet model in rats (Leigh et al., 2020), again suggesting some central selectivity for minocycline. We did not, however, assess for inflammation markers in other peripheral tissues that are involved in energy balance and glucose homeostasis and known to become inflamed with HFD, such as liver.

Previous work indicates that both short-term and long-term HFD exposure induces hypothalamic inflammation in animal models, typically in the ARC (Benani et al., 2012; Cai and Liu, 2011; Waise et al., 2015; Yi et al., 2012). This is marked by a rapid increase in hypothalamic microglia cells, which are thought to be critical regulators of susceptibility of diet-induced obesity (Valdearcos et al., 2017). Previous studies show an increase in neuroinflammation and gliosis in the ARC following only a few days of HFD exposure; however, this acute response temporarily recedes before returning following chronic HFD exposure (Thaler et al., 2012). Other studies report an increase in microglia immunoreactivity in the ARC following 16 weeks of HFD (Yi et al., 2012). It is possible that the 12-week period of HFD used in this study may be in the time window of lessened microglia reactivity in the ARC that may revert to a more reactive state if a longer HFD exposure period was used. One limitation of this study is the focus on microglia reactivity as a marker of neuroinflammation, without examining other inflammatory markers such as astrocyte activity or cytokine levels. While this could be addressed in future studies, minocycline has been shown to be selective for microglia activation and does not directly target astrocytes. Thus, any effects of potential effect of minocycline on astrocyte activity in our study would likely be secondary to microglia effects.

The data here show increases in reactive microglia and upregulation of gene networks related to autophagy in the PVN of the hypothalamus. The PVN is an important hypothalamic subregion that relays ARC signaling to downstream effector regions that regulate autonomic and peripheral nervous system signaling to modulate end organ function. Signals from anorexigenic neurons in the PVN have been shown to decrease satiety and increase food intake, thus contributing to the development of obesity and diabetes (Rahman et al., 2018). Additionally, signaling in this region modulates food intake, energy expenditure, insulin secretion, and glucose metabolism (Long et al., 2015; Rahman et al., 2018; Ma et al., 2020), however no differences in food intake by minocycline were noted in our experiments. Our RNAseq data also suggest that HFD modulates genes regulating insulin signaling in the PVN. We did not verify RNAseq data via PCR, which is a potential limitation of these studies. Emerging research, however, supports the concept that RNAseq results are robust and do not require independent verification as there is a high level of consistency between RNAseq and PCR approaches (Everaert et al., 2017; Coenye, 2021). Overall, our findings suggest the PVN may be an important mediator of HFD-induced inflammation effects on metabolic function, which may be targeted by future therapeutics.

In conclusion, the results from the present study suggest a potential beneficial effect of minocycline on HFD-induced weight gain and insulin resistance via a reduction in inflammatory processes, in this case inflammation in the hypothalamic PVN, independent of food intake. It is important to note these studies were conducted in male mice and sex differences were not explored. While our previous work showed that female mice develop a similar obese phenotype as males in response to HFD (White et al., 2019), whether sex differences exist in terms of hypothalamic neuroinflammatory pathways or responses to minocycline treatment in obesity remain unclear and need to be examined in future studies. Additionally, since younger mice were used in these studies, further work will be needed to determine if aging may also similarly impact neuroinflammation in brain regions related to insulin and glucose homeostasis, and if minocycline may help alleviate age related deficits-with and without obesity as a contributing factor.

## Data Availability

The datasets presented in this study can be found in online repositories. The names of the repository/repositories and accession number(s) can be found below: https://www.ncbi.nlm.nih.gov/geo/, GSE198437.
