# Supplementary figures and images for: Minocycline Reduces Hypothalamic Microglia Activation and Improves Metabolic Dysfunction in High Fat Diet-Induced Obese Mice
Source: Front Physiol. 2022 Jun 16;13:933706. doi: 10.3389/fphys.2022.933706 (PMC9244633; doi:10.3389/fphys.2022.933706)

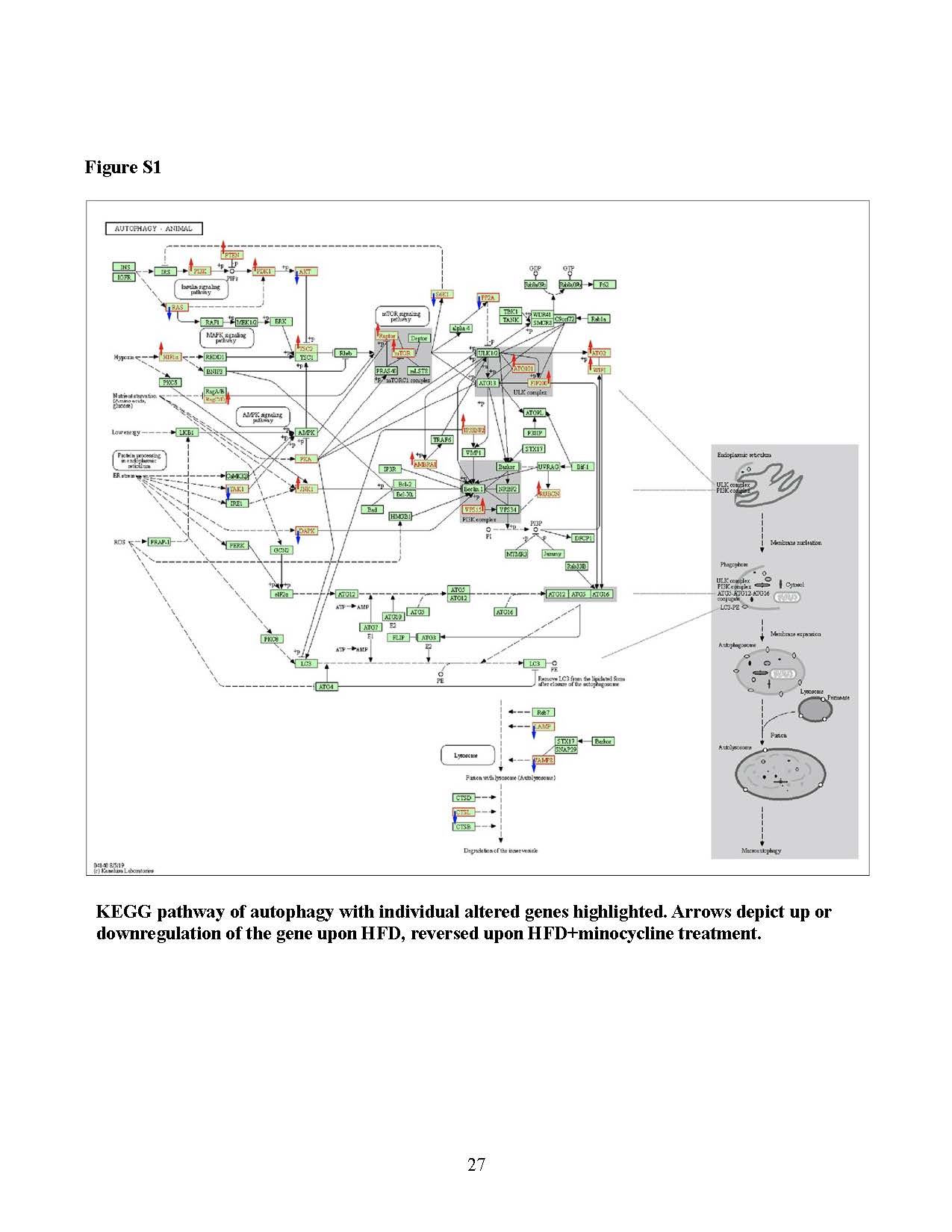

Supplement: Supplementary file 1 [file Image1.JPEG]

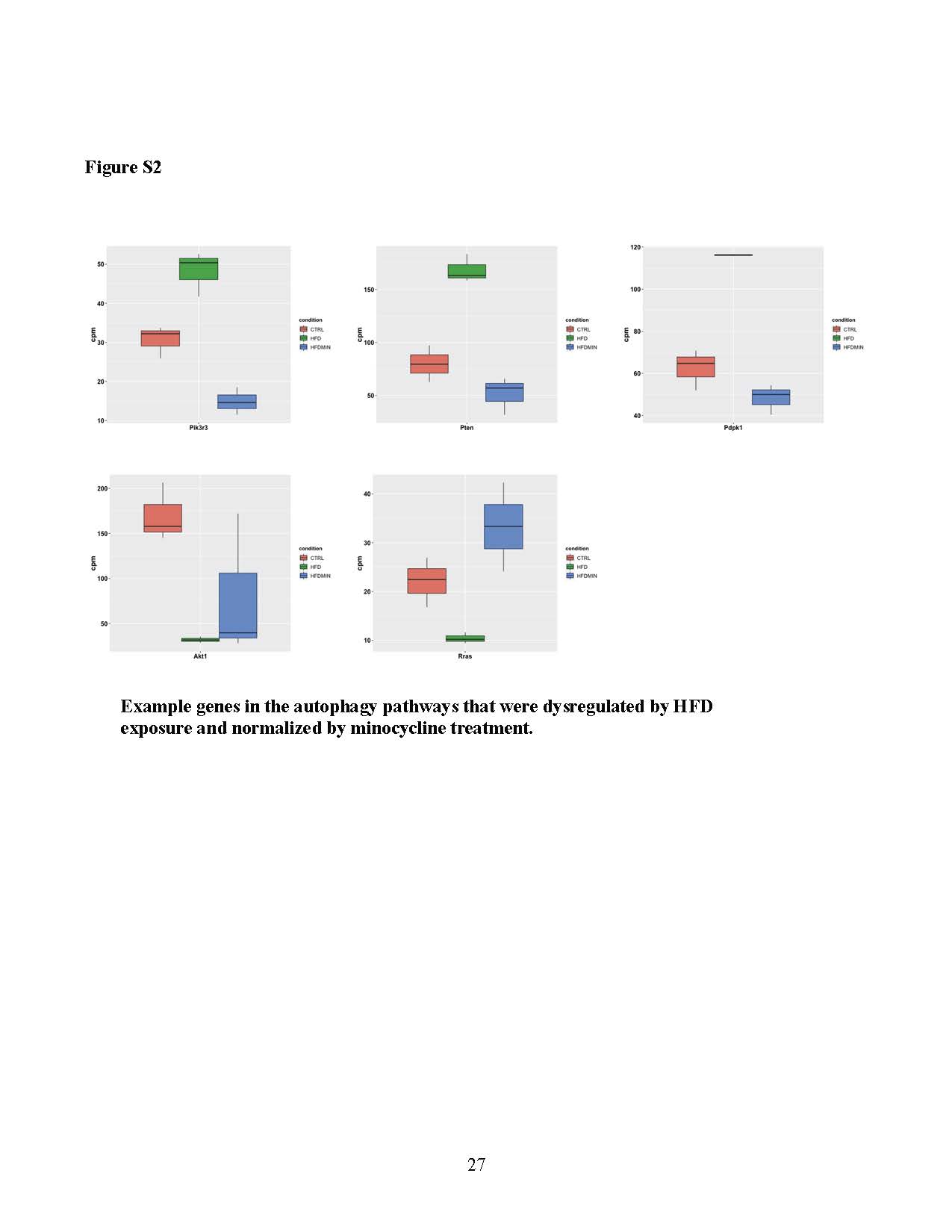

Supplement: Supplementary file 2 [file Image2.JPEG]
